# What compound should I make next? Using Matched Molecular Series for prospective medicinal chemistry

**DOI:** 10.1186/1758-2946-6-S1-O5

**Published:** 2014-03-11

**Authors:** Noel M O’Boyle, Roger Sayle, Jonas Boström

**Affiliations:** 1 NextMove Software, Cambridge CB4 0EY, UK; 2 AstraZeneca, Mölndal, Sweden

## 

A Matched Molecular Pair (MMP) is a pair of compounds which differ only by a well-defined structural transformation [1,2]. Together with large-scale mining of activity or physicochemical data, matched molecular pair analysis (MMPA) has the potential to aid the design of molecules with improved properties by highlighting favourable transformations.

Here we greatly enhance the performance of MMPA for activity prediction by extending to Matched Molecular Series [3,4]. While matched pair transforms are typically equally likely to increase activity as decrease it, series of length 3 or more exhibit a much greater preference for a particular activity order. One possible reason for this is that longer series correspond to more and more specific protein environments, while matched pair analysis often suffers from being an average effect.

**Figure 1:**
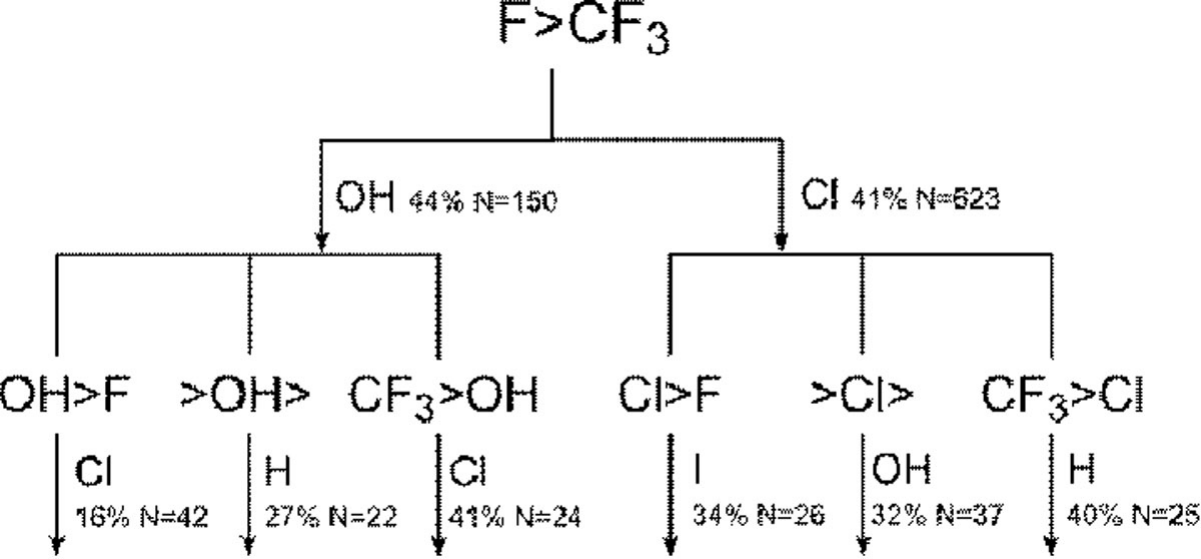
Observed ordering of activities for a matched series.

It will be shown that it is possible to predict, with a known degree of accuracy, what R group should increase/decrease the activity of interest, given an observed ordering of activities for a matched series (Figure 1). Predictions are wholly knowledge-based and interpretable.
